# Electron work functions of ferrite and austenite phases in a duplex stainless steel and their adhesive forces with AFM silicon probe

**DOI:** 10.1038/srep20660

**Published:** 2016-02-12

**Authors:** Liqiu Guo, Guomin Hua, Binjie Yang, Hao Lu, Lijie Qiao, Xianguo Yan, Dongyang Li

**Affiliations:** 1 Department of Chemical and Materials Engineering, University of Alberta, Edmonton, Alberta, Canada T6G 2V4; 2 Corrosion and Protection Center, Key Laboratory for Environmental Fracture (MOE), University of Science and Technology Beijing, Beijing 100083, People’s Republic of China; 3 School of Mechanical Engineering, Taiyuan University of Science and Technology, Taiyuan, Shanxi, 030024, People’s Republic of China

## Abstract

Local electron work function, adhesive force, modulus and deformation of ferrite and austenite phases in a duplex stainless steel were analyzed by scanning force microscopy. It is demonstrated that the austenite has a higher electron work function than the ferrite, corresponding to higher modulus, smaller deformation and larger adhesive force. Relevant first-principles calculations were conducted to elucidate the mechanism behind. It is demonstrated that the difference in the properties between austenite and ferrite is intrinsically related to their electron work functions.

Considerable efforts have been made to correlate the electron work function (EWF) with the adhesive behavior of materials^1^,^2^,^3^,^4^,^5^, which is related to the attractiveness of a surface and characterized by the adhesive force bewteen the surface and its counterface, e.g., the tip of atomic force microscope (AFM) or a surface force apparatus that is used to measure the adhesive force. The adhesive behavior is of importance to understanding of mechanisms responsible for many surface phenomena and processes, e.g., adsorption, friction, contamination, and surface segregation, etc^6^,^7^. EWF reflects the electron activity and is intrinsically related to the surface adhesive behavior^2^,^3^,^4^,^5^,^8^. However, clear correlation between the work function and the adhesive behavior has not been fully clarified, since EWF depends on the type of material and crystallographic planes as well as the surface condition, influenced by surface finishing, adsorption and oxide film, etc. Thus, observations reported in the literature are not always consistent. It has been demonstrated that for single crystals, closely-packed crystallographic planes (low-index) have higher EWFs and lower adhesive force than high-index planes, ascribed to their lower surface electron activity associated with lower surface energy^3^. Such relationship is also true for ordered and disordered structures, e.g., disordered grain boundaries have lower work function with larger adhesive force than crystalline grains^8^,^9^. However, the conclusion of higher EWF corresponding to lower surface energy and thus lower adhesive force is contradictory to some reported studies including the observations reported in this article. Thus, one of main objectives of this work is to understand and clarify the discrepancy.

The Kelvin probe is widely used to measure work function. However, its probe with a tip size on mm scale collects signals from a relatively large area, which could be influenced by unexpected factors. With the development of multifunctional scanning probe microscopy (SPM), mapping local work function and evaluating corresponding properties, e.g., adhesive force and modulus, can be carried out. This makes it achievable to establish more precise relationships between EWF and the material properties.

For the present study, a duplex stainless steel is selected as a sample material, which consists of ferrite (α) and austenite (γ) phases. In this study, work functions, adhesive forces and moduli of the two phases were measured using Peak Force KPFM in order to determine the relationship between the work function and adhesive force on micro-scale with the objectives of 1) investigating these properties of ferrite and austenite, and 2) clarifying the discrepancy in relevant studies and observations reported in the literature. In parallel, *ab initio* calculations were conducted to confirm the experimental results and understand mechanisms behind.

## Results and Discussion

**Figure 1:**
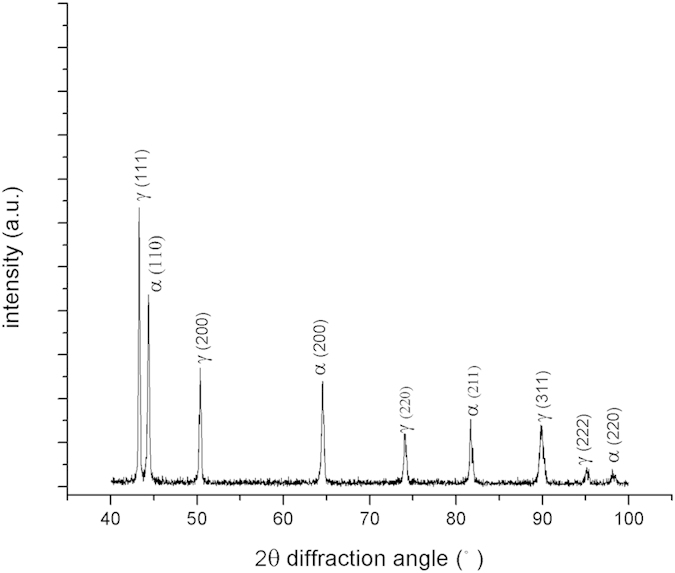
A X-ray diffraction pattern of 2507 duplex stainless steel.

**Figure 2:**
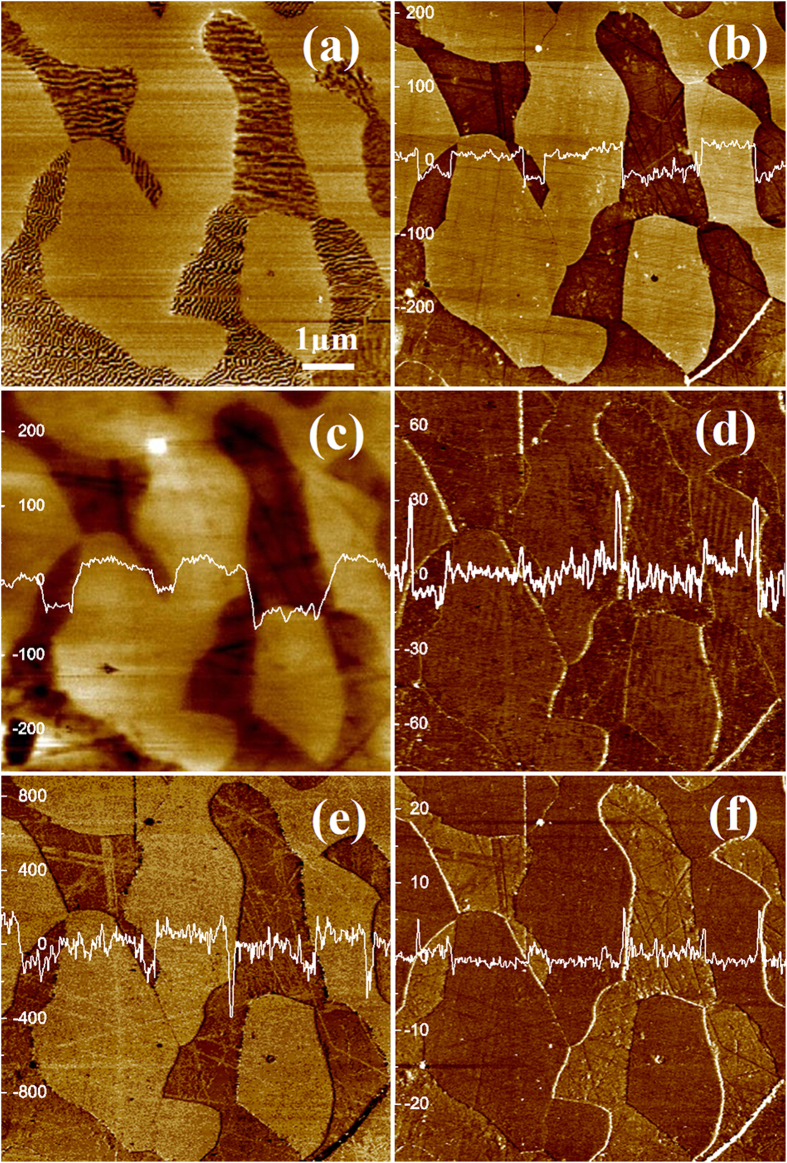
(**a**) A MFM image of the duplex stainless steel; (**b**) An AFM topography image and with a line profile of height; (**c**) Work function mapping with a potential profile; (**d**) Adhesion mapping with an adhesion profile in nN; (**e**) Modulus (GPa) mapping; and (**f**) Deformation mapping (nm).

A XRD pattern of the duplex stainless steel is presented in Fig. 1, which confirms that the steel consisted of austenite and ferrite phases. For AFM analysis, MFM (magnetic force microscopy) was employed to identify the ferrite and austenite phases thanks to their distinctive magnetic characteristics. A MFM image is illustrated in Fig. 2(a). As shown, the ferrite phase has a striped appearance due to its ferromagnetic behavior, while the paramagnetic austenite phase shows a uniform appearance. Corresponding topography (Fig. 2(b)) of the same area reveals that austenite (lighter) is higher than ferrite (darker), which is consistent with previous work^10^,^11^,^12^. The difference in height is caused by the electrochemical polishing during which the ferrite phase dissolved faster than austenite due to its relatively lower corrosion resistance.

Figure 2(c–f) present maps of local work function, adhesion, modulus and deformation of the same areas. One may see that the austenite has higher EWF and larger adhesive force than the ferrite. Besides, the austenite shows a greater modulus and smaller deformation than the ferrite. Average values of the properties are presented in Table 1. The dependence of the mechanical strength on EWF is in agreement with previous studies^13^,^14^, i.e., the higher the work function, the greater the modulus. As demonstrated previously, Young’s modulus of metals increases as work function increases in a sixth power relationship^13^. An increase in EWF reflects a higher stability of valence electrons, leading to increased mechanical strength and enhanced resistance to corrosion attack, respectively. These happen because a more stable electron state corresponds to a raised degree of confinement to valence electrons, which strengthens the metallic bonding and limits valence electrons to participate in electrochemical reactions.

Regarding the correlation between work function and adhesive behavior, it is appropriate to correlate EWF with the adhesive force via surface energy as a bridge. A surface with a larger surface energy is more reactive or more attractive, corresponding to a larger adhesive force when in contact with or approaching a counter-face. According to reported experiments and theoretical analyses using *ab initio* method and a stabilized jellium model on the surface energy and work function^1^,^2^, higher EWFs correspond to larger surface energies. This agrees with our current observations that the austenite exhibited a larger adhesive force and higher EWF than the ferrite. Theoretically, we may estimate the ratio of average EWF of austenite to that of ferrite. For metallic materials, EWF is proportional to 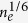 15, where 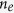 is the density of valence electrons. Austenite has a closely packed fcc structure which has a higher 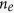 than the loosely packed bcc ferrite crystal. This renders austenite to possess a higher work function than ferrite, which can be seen from the ratio of 
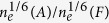
, estimated as the ratio of atomic packing density of austenite to that of ferrite, i.e.,


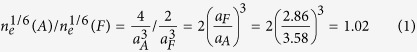


where 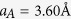 and 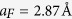
16. This ratio is consistent with that of measured work functions of austenite and ferrite (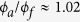, see Table 1). As a result, the austenite should exhibit a larger adhesive force than ferrite. This is again consistent with the observed relationship between the work function and adhesive force for ferrite and austenite.

**Figure 3:**
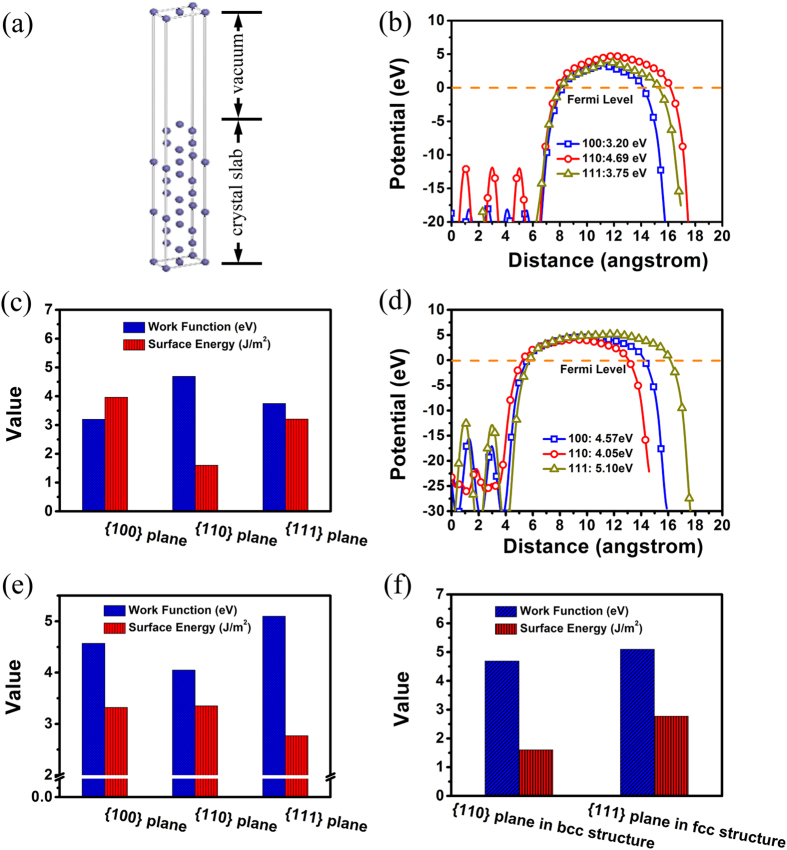
(**a**) An atomistic model for surface property calculation; (**b**) Potential distributions across surface (100), (110) and (111) planes of ferrite bcc structure, respectively; (**c**) The relation between work function and surface energy for different planes of ferrite bcc structure; (**d**) Potential distributions across surface (100), (110) and (111) planes of austenite fcc structure, respectively; (**e**) The relation between work function and surface energy for different planes of austenite fcc structure; (**f**) Work functions and surface energies of the most stable planes of (110) of ferrite bcc and (111) of austenite fcc structures, respectively.

*Ab initio* calculations were employed to analyze the work function, surface energy, adhesion and modulus in order to understand the experimental observations. Figure 3(a) illustrates an atomistic configuration for surface property calculation, which consists of a crystal slab and vacuum layer. Table 2 gives calculated equilibrium lattice constants, elastic properties, Poisson ratios, electron work functions and surface energies of bcc ferrite and fcc austenite, respectively. In Fig. 3(b), potentials across different surface planes for ferrite are plotted, from which EWFs of three low-index crystal planes were determined, which follow the order of 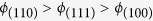. In Fig. 3(c), EWFs and surface energies of the three crystallographic planes are illustrated. As shown, the crystallographic planes exhibit a reversed order of their surface energies, i.e. 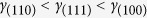. Figure 3(d) presented the potentials across different surface planes for the fcc austenite structure. As shown, three low-index planes have their EWFs in the order of 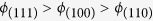. The order of corresponding surface energies is also reversed as Fig. 3(e) illustrates i.e. 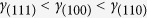. According to the calculation, crystal planes of a specific crystal with lower surface energies (more inert) have higher EWFs, corresponding to lower adhesive forces due to their lower activities. Such general relationships between crystallographic plane orientation planes and surface properties including EWF and adhesive force are consistent with experimental studies^4^, which confirm that the higher EWF of a crystal plane corresponds to a lower adhesive force. Such a relationship between EWF and adhesive force is applicable to ordered and disordered domains in a material as well. For instance, crystal grains have higher EWF and smaller adhesive force in contrast with disordered grain boundaries which show lower EWF and larger adhesive force^8^.

However, the situation changes when look at different crystals or materials such as austenite (A) and ferrite (F). It is of interest to compare their thermodynamically stable planes, which are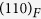 and 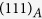, respectively (see Fig. 3(c,e)), having minimal surface energy and maximal EWF. As shown in Fig. 3(f), 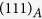 plane of austenite has a higher EWF and larger surface energy than ferrite 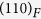 plane. Or in other words, comparing austenite and ferrite, the former has a higher EWF and correspondingly larger adhesive force. This is consistent with the results of experimental measurements reported in Table 1. It is clear that the relation between EWF and adhesive forces for the different phases or materials is different from that for different crystallographic planes of a specific crystal or the same material as illustrated in Fig. 3(c,e).

The above-mentioned difference often results in confusion or discrepancy, which is however explainable through analyzing the correlation between EWF and surface energy or interfacial energy. Comparing austenite with ferrite, the stronger bond strength of austenite makes austenite’s surface is more attractive with stronger broken bonds when a surface is created. This leads to higher surface energy and higher EWF as well, since it is less easy to take electrons from the stronger broken bonds on surface. Such a surface has higher attractiveness, which results from its tendency to attract charges from the surrounding media, leading to stronger electrostatic interactions. However, the situation changes for different crystallographic planes of a single crystal. In this case, the closely packed plane has the minimum surface energy and its electrons are in the most stable state, resulting in the highest EWF and least adhesive force. A more attractive crystallographic plane has a lower EWF, corresponding to a lower barrier for electrons to escape. Thus, the higher attractiveness of a crystallographic plane with a lower EWF may result in a higher mobility of electrons or a higher degree of freedom for electrons to react with the surrounding media. As for grain boundaries (GB), the situation is similar. This disordered region with defects has a higher energy and is more reactive than crystalline zones (grains)^8^. Electrons are active and easier to be extracted from GB. Thus, GBs exhibit larger adhesive force and lower EWF.

From the above analysis, the adhesive behavior is governed by energy, e.g., surface energy, and correlated with EWF in an indirect way because electrons in different types of material behave differently. It would be helpful to have a further look at the correlation between EWF and surface energy in order to prevent misinterpretation of observed EWF- adhesion relationships reported in the literature. For instance, Using the *ab initio* method, Hugosson *et al.*^1^ calculated surface energies, surface electronic structures and work functions for the (100) surface of 3d (Sc–Cu), 4d (Zr–Ag) and 5d (La–Au) transition metal carbides. Their calculations indicate that higher EWF correspond to lower surface energy, which is opposite to results for metals. Such difference should be ascribed to different characteristics of atomic bonds in ceramics compared to metallic bonds. Atoms in ceramic materials are connected by covalent bonds or mixture of covalent and ionic bonds, in which electrons are generally localized. When a surface is created and atomic bonds are broken, electrons are confined by the atoms to which they originally belong. Or in other words, the broken bonds are not active. Thus, the higher the surface work function, the more stable the surface accompanied with lower surface energy. For metallic materials, when a surface is created with formation of broken bonds^17^, these broken metallic bonds are however active and electrons participate in the electrostatic interactions with the surrounding medium to form, e.g., an adsorption layer. In this case, the higher EWF associated with stronger atomic bonds leads to a higher surface energy. For a single crystal, its closely packed crystallographic plane with the lowest surface energy has the smallest broken-bond density and the majority of atomic bonds are in the surface plane^17^. This raises the difficulty to extract electrons from the closely-packed plane, thus elevating its EWF with lowered surface energy.

## Conclusions

In summary, with local electron work function mapping and property evaluation using a multimode AFM, we analyzed work functions, adhesive forces, moduli and deformation behaviors of austenite and ferrite phases in a duplex stainless steel. It was demonstrated that austenite had higher EWF, larger adhesive force, and higher modulus, compared to ferrite. The higher values of these properties of austenite are attributed to its larger valence electrons density, compared to that of ferrite. The experimental observations are consistent with first-principle calculations.

In addition to the specific comparison between austenite and ferrite, this study is also intended to help clarify the discrepancy in the literature, which has become explainable with the following general statements:

1. For different phases or domains and structures of metallic materials, the higher the EWF, the larger the surface energy and thus the larger the adhesive force. Larger EWF corresponds to stronger broken bonds on surface.
2. For different crystallographic planes of the same crystal, the higher the EWF, the lower the surface energy and thus the smaller the adhesive force.
3. The relation between EWF and adhesive force should be influenced by the type of material, which may depend on whether the surface broken bonds are active or not, i.e., the surface electrons are active (metallic) or are confined by nuclei to which they originally belong (e.g., ceramic).

## Methods

### Sample preparation

The material under study is a conventional 2507 duplex stainless steel^10^,^18^. Specimens cut from the steel were wet ground with SiC paper up to 2000 grit, and then mechanically polished using a 1.5 μm-diamond paste. The sample surface was electrochemically polished in a mixed solution of HNO_3_:H_2_O = 1:1 for 20 sec under an applied voltage of 1.2 V. The electrochemically polishing facilitated distinguishing the ferrite and austenite phases. The specimens were ultrasonically cleaned in ethanol and dried by a N_2_ gas flow.

### X-ray diffraction

For self-containing information, X-ray diffraction analysis was made to determine phases in the steel using a Siemens diffractometer (D5005) with Cu *Ka* radiation.

### Atomic force microscopy

Experiments were performed using a Bruker MultiMode atomic force microscope-AFM 8 with PeakForce KPFM capability. The experiments were performed right after the samples were electro-polished. Bruker magnetic probes (MESP) with its force constant of 2.8 N/m were used for MFM imaging and work function measurements, while Bruker ScanAsyst-Air probes (silicon) with a force constant of 0.4 N/m were used to measure the adhesive force and diamond probes with a force constant of 350 N/m were used to measure modulus and deformation. It should be indicated that the MESP used to measure work function has a force constant of 2.8 N/m, which is not suitable for measuring mechanical properties of duplex stainless steel. Thus, the softer Bruker ScanAsyst-Air and stronger diamond probes were used to determine the local mechanical properties that correspond to the local EWF. The measurements were performed with changed probes (controlled by the AFM system) in the same mode, i.e. Peak Force KPFM, for the same area under analysis.

### *Ab initio* calculations

The first-principles calculations were implemented with an ABINIT package^19^,^20^, which allows calculations of the total energy, charge density and electronic structure of systems within Density Functional Theory (DFT), using pseudopotentials. Projector augmented-wave (PAW) pseudo-potentials^21^ and Perdew- Burke-Ernzerhof Generalized Gradient Approximation (GGA) of exchange-correlation functional^22^ were adopted for the calculations. In order to calculate elastic properties, an energy cutoff of 12 hartree (1 hartree = 27.211eV) and a 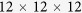 k-point mesh were used to achieve self-consist field convergence with the tolerant potential residual V(r) less than 10^−12^ hartree. According to the definition of elastic constant, which is the second derivative of total energy with respect to the strains, elastic properties were calculated by means of the relation between total energies and specific applied strains. Three specific strains were set up for the calculation of independent elastic constants of fcc and bcc structures^23^. Bulk modulus, shear modulus, and Young’s modulus of polycrystalline metals were calculated according to the Voight–Reuss–Hill bounds^24^. For the surface-property calculation, a larger supercell was set up in which a vacuum layer about 1nm thickness was created over the crystal system. Atoms in the first surface layer were relaxed and the tolerance on maximal force for the surface relaxation was 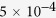 hartree/Bohr. A 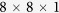 k-point mesh was used to achieve self-consist field convergence. Based on the DFT electronic calculation, electron work function 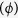 was extracted from the difference between the value of the electrostatic potential in vacuum and the Fermi energy level: 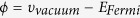, where 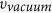 is the electrostatic potential in vacuum and 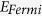 is the Fermi energy level, 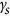 is the surface energy calculated as: 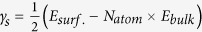, where 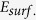 was the energy of system consists of crystal slice and vacuum layer, 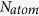 was the atom number in the system, 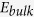 was the energy of each atom in bulk state.

## Additional Information

**How to cite this article**: Guo, L. *et al.* Electron work functions of ferrite and austenite phases in a duplex stainless steel and their adhesive forces with AFM silicon probe. *Sci. Rep.* **6**, 20660; doi: 10.1038/srep20660 (2016).

## Figures and Tables

**Table 1:** Measured average values of electron work function (EWF), adhesive force (with AFM Si probe), modulus and deformation of ferrite and austenite of the duplex stainless steel.

	EWF (eV)	Adhesive force (nN)	Modulus (Gpa)	Deformation (nm)
Ferrite	4.953	10.62	163.8	2.244
Austenite	5.045	12.53	182.2	1.185

**Table 2:** Calculated equilibrium lattice constant (a), elastic constants (C_ij_), bulk modulus (B), shear modulus (G), Young’s modulus (E), electron work function (EWF), Poisson’s ratio and surface energy of ferrite bcc structure and austenite fcc structure of the duplex stainless steel.

	a (A)	C_11_(GPa)	C_12_(GPa)	C_44_(GPa)	B(GPa)	G(GPa)	E(GPa)	Poisson’s ratio	EWF (eV)	Surf. energ(J/m^2^)
Ferrite	2.829	286.9	163.9	110	204.9	87.2	229	0.313	(100): 3.2	(100): 3.96
	2.866^a^	226^a^	140^a^	116^a^	166^b^	80^b^	208^b^	0.291^b^	(110): 4.69	(110): 1.6
									(111): 3.75	(111): 3.20
Austenite	3.455	419.4	213.2	237.7	281.9	170	424.6	0.25	(100): 4.57	(100): 3.32
	3.647^a^								(110): 4.05	(110): 3.35
									(111): 5.10	(111): 2.77

Note: superscript.

^a^Data from ref. 25.

^b^data from ref. 26.
